# The challenges and complications of re-implantation of the penis following amputation

**DOI:** 10.4314/gmj.v56i1.9

**Published:** 2022-03

**Authors:** Moumita De, Rakesh Dawar

**Affiliations:** 1 All India Institute of Medical Sciences, New Delhi-110029, India

**Keywords:** Penile amputation, Microsurgery, Replantation

## Abstract

Amputation of the penis is a rare and devastating injury. The etiologies vary from accidental, self-inflicted to attacks due to sexual jealousy and revenge. In the present era of microvascular surgery, replantation is the standard care. However, replantation of the penis comes with its own set of difficulties and complications. Knowledge of the anatomy and prior knowledge of the possible complications makes the surgeon aware of the course of events after a replantation. It helps in devising strategies to overcome these challenges methodically. We present a case of penile replant with the complications that we encountered, and the measures are taken to counter them on our way to a successful outcome.

**Funding:**

No external funding

## Introduction

Total amputation of the penis is an extremely rare occurrence. In literature, the most common causes cited are self-mutilation, assault, machinery injury, or road traffic injury^1^. Amputation of the penis is associated with great psychological and functional implications^2^. The current standard of treatment is microsurgical replantation in all possible cases. We present a case of total penile amputation at the base of the penis replanted and the challenges we faced to the recovery. We also document the steps taken to face the challenges.

Amputation of phallus carries with it many psychosocial implications. The stigma of mutilated genitalia is often associated with accidents, self-mutilations, sexual jealousy, punishment, and revenge. Obtaining a true and proper history is often difficult because of the associated humiliation on the victim's part.

## Case Report

A 21-year-old man was brought to the emergency room with a total amputation of the penis at the base. The initial history given was accidental trauma, which later on, after a thorough and patient history taking in the post-operative period, was revealed as a crime of passion. The young victim was in a relationship with a transgender person previously. After he decided to terminate the relationship, his partner laced his drink with some sedatives and amputated his penis with some sharp object. The victim woke up in severe pain in a pool of blood. His family members rescued him, and he was taken to a medical facility.

**Figure 1:**
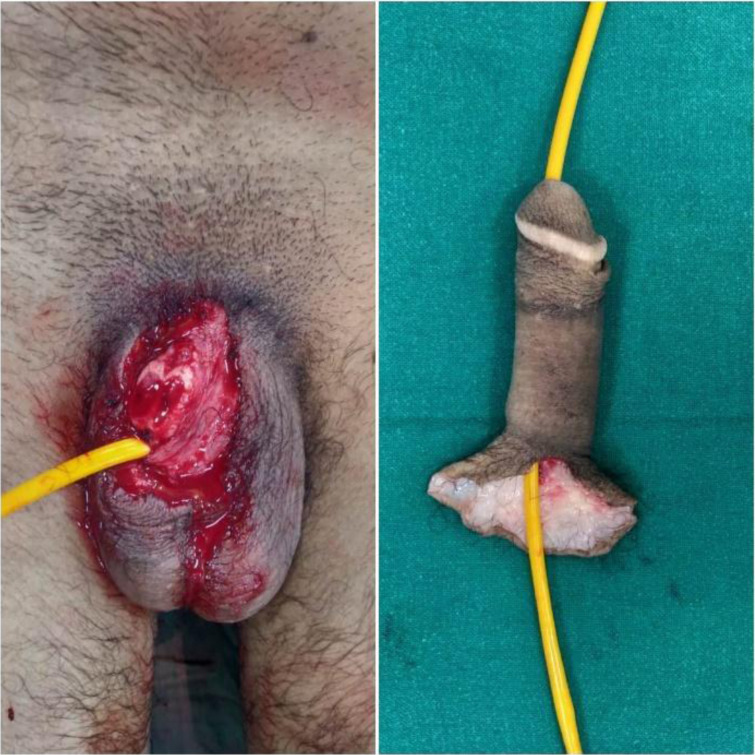
A 12Fr silicone catheter was passed across the urethra of the amputated part and into the urethra of the stump

The victim was initially treated at a local hospital where a Foley's catheter was inserted in the proximal urethra, and compression dressing was done. The amputated part was preserved properly with a warm ischemia time of approximately 6 hrs. The amputated part was immediately taken in the operating room (OR), and dissection was done under a microscope. The patient was hemodynamically stable on arrival. The Trauma Surgery team attended to him. The Vitals on arrival were Pulse rate- 84/min; BP- 124/82 mm of Hg; respiratory rate- 18/min. A primary survey and initial management were done. After a negative skin sensitivity test, the patient was given a dose of tetanus toxoid and a single dose of injection amoxicillin + clavulanate. Routine blood investigations were sent, and blood was sent for cross-matching. After that, the patient was shifted inside the OR. Under general anaesthesia, the proximal stump was explored, and all structures were identified and tagged (Figure 1). A 12 Fr silicone catheter was passed across the urethra of the amputated part and into the urethra of the stump. Firstly, the urethra and corpus spongiosum were repaired over the catheter using 8-0 vicryl in an interrupted fashion. Next, the tunica over the cavernosal bodies were repaired on the ventral side (3 O'Clock to 9 O'clock) in a watertight fashion using 5-0 vicryl. This provided the necessary support for the subsequent cavernosal (deep central arteries) arterial anastomosis. Inside the corpora cavernosa, the deep central arteries of the cavernosa were identified and anastomosed with 10-0 nylon sutures.

**Figure 2:**
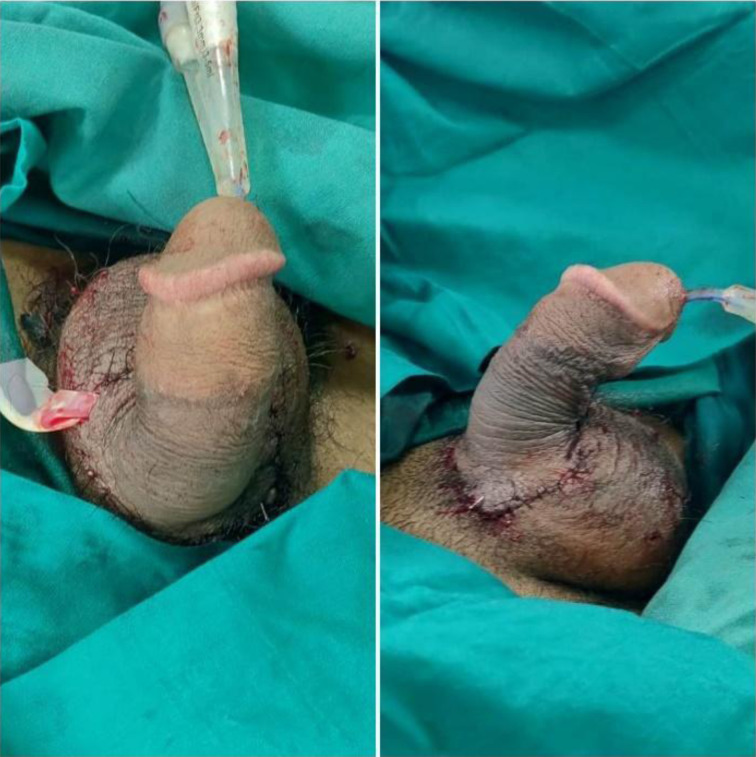
The glans and shaft of the penis with prepuce changed to normal colour, turgor, and capillary refill

The rest of the tunica over the dorsal part (9 O'clock to 3 O'Clock) was closed in a water-tight fashion using 5-0 vicryl. One dorsal artery and the deep dorsal vein were anastomosed with 9-0 nylon sutures. One superficial dorsal vein was also repaired. A pair of dorsal nerves were coapted with 10-0 nylon and fibrin glue. At the end of the procedure, the glans and shaft of the penis with prepuce changed to normal colour, turgor, and capillary refill. (Figure 2) Doppler signal was audible over the dorsal artery at the end of surgery. The patient's vitals were stable throughout the surgery, and no blood transfusion was done as per the advice of the anaesthesia team. The patient was kept on antibiotic coverage for Gram-positive and Gram-negative organisms in the post-operative period. (Amoxicillin + Clavulanate; Amikacin) On post-operative day two, the preputial skin started to show patchy necrotic changes with a collection of hematoma beneath. The hematoma was evacuated bedside. Doppler signal was audible and glans vascularity was excellent. Hence re-exploration was not contemplated at that time.

**Figure 3:**
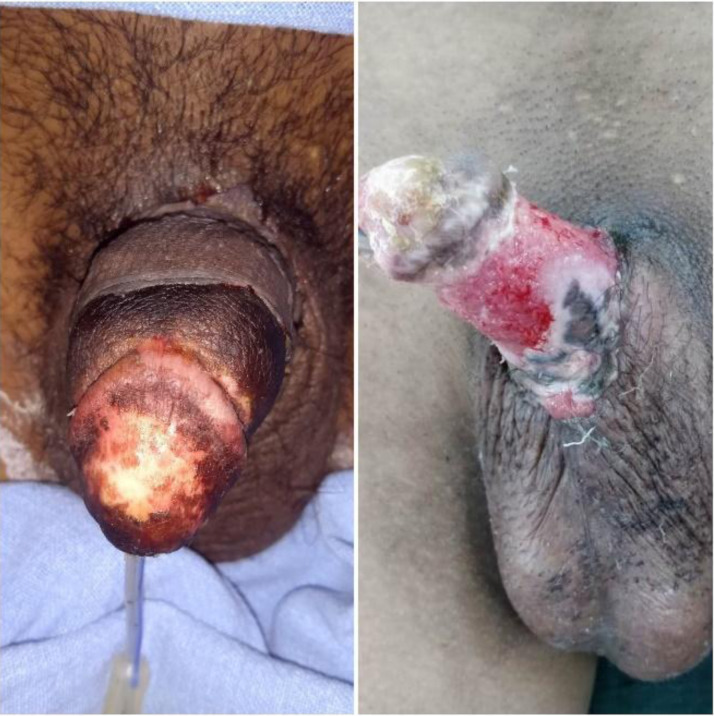
Debridement of all necrotic skin was done

On post-op day 8, there was a sudden cessation of Doppler signal and an increase in the necrosis of the preputial skin. The patient was taken up on an emergency basis to the OR. On exploration, full-thickness necrosis of skin 1cm proximal to corona was present. A thrombus was found in the dorsal artery with absent flow. Debridement of all necrotic skin was done (Figure 3). After the arterial anastomosis was re-done, the glans and shaft vascularity improved with increased blood flow. A thin split-thickness graft was then harvested from his left thigh and applied to the shaft of the penis.

The patient received hyperbaric oxygen therapy in the post-operative period for 14 days. Close monitoring of glans vascularity was done and the first dressing change was done on post-op day 5 following re-exploration.

The patient developed a necrotic patch surrounding the meatus, which gradually formed a well-demarcated eschar. The skin graft initially showed a good take. Later, the graft was lost almost completely. A decision was taken to withhold any immediate surgical procedure, and the patient was put on hyperbaric oxygen therapy with daily saline dressing for the next 14 days. On the 15th post-operative day, the peri-meatal eschar separated by itself to show a healthy granulating small raw area. The penile shaft was covered with healthy granulation tissue by the end of 2nd week. Debridement with a thin split-thickness graft was done for the raw areas. (Figure 4a) All wounds healed uneventfully. The catheter was removed at 6weeks.

**Figure 4a:**
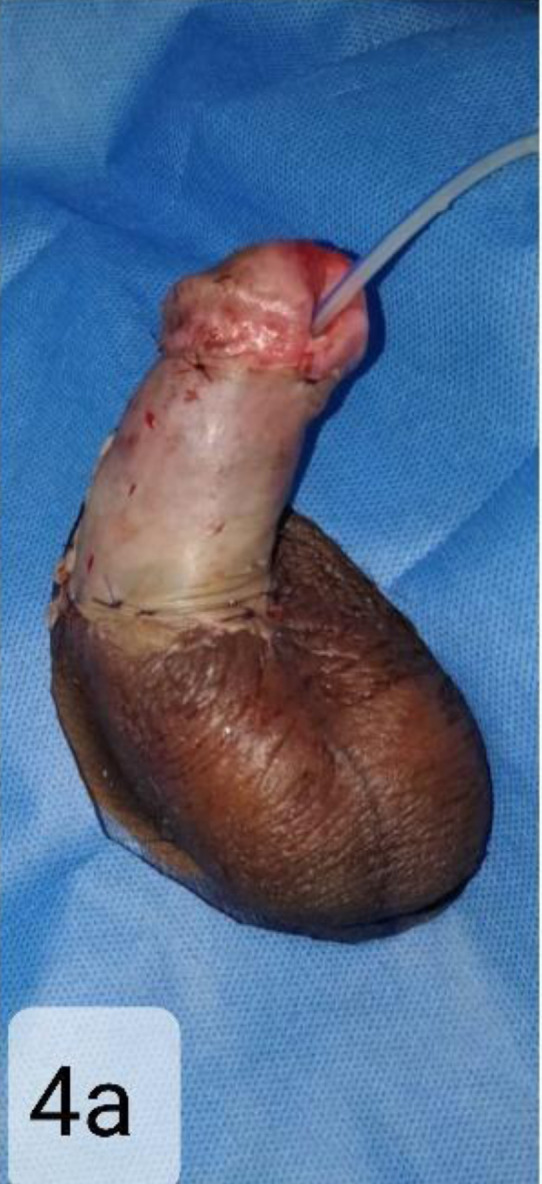
Debridement with a thin split-thickness graft was done for the raw areas. All wounds healed uneventfully.

Follow-up at 10 weeks shows the patient has a good shaft length with stable skin coverage. (Figure 4b) He can void urine in the standing position, although the stream has mild splaying due to slightly wide meatus. He also has some return of sensation over the glans. Erection is not achieved, but he experiences occasional engorgement and apparent enlargement.

**Figure 4b:**
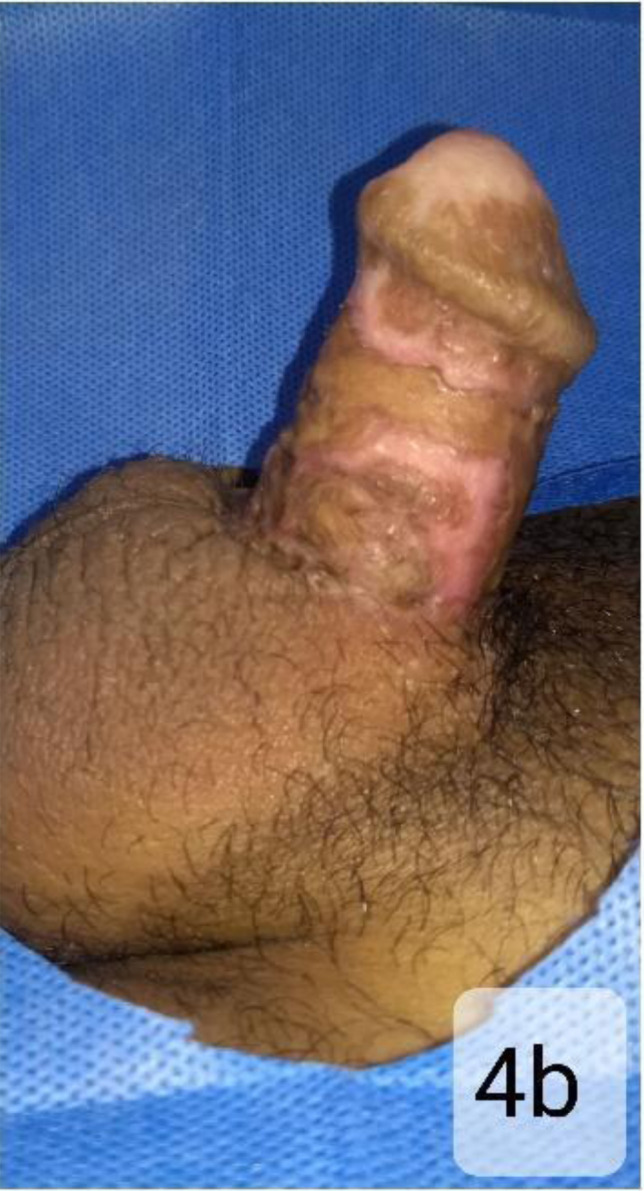
The catheter was removed at 6weeks. Follow-up at 10 weeks shows the patient has a good shaft length with stable skin coverage

The patient is attached to our outpatient clinic as well as that of the Urology Department. Also, he is seen and counselled by a clinical psychologist.

## Discussion

The first cases of microsurgical replantation of the penis were done in 1976 and 1977.^3^,^4^ To date, over 100 cases of penile replantation have been reported. 2. However, all were not repaired by microsurgery. Some of the initial authors reported survival of the penis with non-microsurgical repair with just cavernosal approximation as described by Ehrich back in 1926.^5^ Genital mutilation carries great psychological impact, and as a good number of cases are self-inflicted. A meta-analysis by Morrison et al found that 47.8% of patients have some psychiatric illness;^4^ cases warrant thorough psychological evaluation and treatment.^2^ Also, it is to be emphasized that psychiatric illness is not a contraindication to penile replantation^2^, and Fujiki et al reported successful second replantation in a schizophrenic patient.^6^

In our case, the victim was attacked by persons known to him. The challenges in our case were,

- Prolonged warm ischaemia time of 6 hours at the time of presentation. Being a tropical country, 6 hours in the summer months is significant. The patient was taken to OR almost 12 hrs post-injury.
- Injury at the base- so there was no place to put a tourniquet. Control of bleeding was always an issue.
- Difficult localisation of deep artery of the penis within the cavernosal sinusoids

While Wei FC et al. l reported successful replantation after 16 hrs of warm ischaemia time^7^, Riyach et al. states that the standard 6 hours warm ischaemia time can be stretched even up to 24 hrs.^8^The challenges of a proximal amputation were tackled with meticulous and fast dissect ion with identification and clamping of all the dissected vessels till anastomosis was performed.

The post-operative period was a roller coaster ride for both the patient and the surgeons as we experienced different complications along the way to recovery. The initial complication of preputial necrosis was treated with debridement and grafting. Preputial necrosis is stated to be the commonest complication.^4^ In the pre-microvascular era, it was suggested that the prepuce should be removed at the primary procedure as sloughing of the foreskin is almost expected^9^, Tuffaha et al. went ahead to state necrosis of penile skin as an unavoidable process with their cadaveric studies on the importance of the external pudendal system.^10^ While early detection of loss of flow in the dorsal artery was crucial in the salvage of the organ, and Henrikkson et al reported salvage by re-anastomosis after 15 hours^11^, such delayed thrombosis as post-operative Day 8, as seen in our case is not reported before. Vigilant clinical and Doppler monitoring by our residents played the most significant role. While thrombectomy and re-anastomosis were successful, unexpected delayed graft loss, aggravated by perimeatal necrosis posed new challenges. We believe at this point hyperbaric oxygen therapy played a role as previously experienced by other authors as well^1^,^12^, and we were able to finally salvage the organ. While Li GH et al advocated a suprapubic cystostomy in all cases^13^, our patient was managed on per-urethral silicone catheter only which was removed on 6th wk.

## Conclusion

Penile amputation is an unusual injury. We may see very few cases throughout our careers.

So, there can be multiple learning points from one such case. Multiple anastomoses, neural coaptation, timely re-exploration, and hyperbaric oxygen therapy were crucial in this case. Also, patient confidentiality and post-operative counselling play a crucial role in such cases.
